# CD1d^hi^PD-L1^hi^CD27^+^ Regulatory Natural Killer Subset Suppresses Atopic Dermatitis

**DOI:** 10.3389/fimmu.2021.752888

**Published:** 2022-01-05

**Authors:** Keun Young Min, Jimo Koo, Geunwoong Noh, Dajeong Lee, Min Geun Jo, Ji Eon Lee, Minseong Kang, Seung Yeun Hyun, Wahn Soo Choi, Hyuk Soon Kim

**Affiliations:** ^1^ Department of Immunology, School of Medicine, Konkuk University, Chungju, South Korea; ^2^ Department of Allergy, Allergy and Clinical Immunology Center Cheju Halla General Hospital, Jeju, South Korea; ^3^ Department of Health Sciences, The Graduate School of Dong-A University, Busan, South Korea; ^4^ Department of Biomedical Sciences, College of Natural Science, Dong-A University, Busan, South Korea

**Keywords:** regulatory natural killer cells (NKreg), transforming growth factor (TGF)-β, atopic dermatitis (AD), group 2 innate lymphoid cells (ILC2s), T helper 2 (T_H_2) cells

## Abstract

Effector and regulatory functions of various leukocytes in allergic diseases have been well reported. Although the role of conventional natural killer (NK) cells has been established, information on its regulatory phenotype and function are very limited. Therefore, the objective of this study was to investigate the phenotype and inhibitory functions of transforming growth factor (TGF)-β-producing regulatory NK (NKreg) subset in mice with MC903-induced atopic dermatitis (AD). Interestingly, the population of TGF-β-producing NK cells in peripheral blood monocytes (PBMCs) was decreased in AD patients than in healthy subjects. The number of TGF-β^+^ NK subsets was decreased in the spleen or cervical lymph node (cLN), but increased in ear tissues of mice with AD induced by MC903 than those of normal mice. We further observed that TGF-β^+^ NK subsets were largely included in CD1d^hi^PD-L1^hi^CD27^+^ NK cell subset. We also found that numbers of ILC2s and T_H_2 cells were significantly decreased by adoptive transfer of CD1d^hi^PD-L1^hi^CD27^+^ NK subsets. Notably, the ratio of splenic Treg per T_H_2 was increased by the adoptive transfer of CD1d^hi^PD-L1^hi^CD27^+^ NK cells in mice. Taken together, our findings demonstrate that the TGF-β-producing CD1d^hi^PD-L1^hi^CD27^+^ NK subset has a previously unrecognized role in suppressing T_H_2 immunity and ILC2 activation in AD mice, suggesting that the function of TGF-β-producing NK subset is closely associated with the severity of AD in humans.

## Introduction

Atopic dermatitis (AD) is known as a chronic inflammatory skin disease. It is referred to as atopic eczema with typical symptoms such as itchy, red, swollen, and cracked skin lesions. Although AD is widespread and on the rise in developed countries, the exact pathological mechanism of AD is not fully understood yet (1–3). Currently, it is a typical type 2 helper T (T_H_2) cell-mediated hypersensitive immune disorder in which various immune cells are known to participate in the development of skin inflammation (4). In AD, T_H_2 cells secrete IL-4, IL-5, and IL-13 known to promote allergic responses (5, 6). These T_H_2 cytokines can stimulate IgE production from B cells, activate mast cells, and lead to infiltration of eosinophils or other immune cells into inflamed tissues (7). Recent studies have also reported that type 2 innate lymphoid cells (ILC2) have the function of T_H_2 cells in peripheral tissues. ILC2 is also well known to initiate and participate in T_H_2 cell-mediated responses by secreting T_H_2 signature cytokines such as IL-5 and IL-13 in peripheral tissues (8, 9).

Natural killer (NK) cells are well known as a type of anti-microbial lymphocytes in innate immunity (10, 11). NK cells can release proteolytic enzymes and interferon (IFN)-γ to remove virus-infected cells, intracellular bacteria, and tumor cells (12, 13). NK cells can be classified into several subsets, depending on the profile of cytokine secretion and their function (14). Typically, NK1 cells (also called conventional NK cells) secrete IFN-γ and NK2 cells secrete T_H_2 cytokines (15–18). Subsets of IL-17 secretion (NK17) or IL-22 secretion (NK22) NK cells have also been reported (19–21). Compared to the past classification of the NK subset, various innate lymphoid cell types have been recently introduced, and in particular innate lymphoid cells (ILCs) have been proposed as representative helper innate immune cells (22). In addition, various T cell-lineage subsets have recently been defined in innate T cells such as NKT or γδ T cells that partially share receptors with conventional NK cells (23, 24). Therefore, it is necessary to more clearly distinguish the classification of NK. Accumulating evidences have demonstrated that some types of NK cells have a suppressive function like regulatory T (Treg) cells by secreting IL-10 or TGF-β in transplantation, pregnancy, and some infections (25–30). Although the role of NK cells in allergic diseases including AD is poorly understood, recent studies have reported that the number of circulating NK cells in blood samples of AD patients are generally decreased, but increased in inflammatory skins (31–34). However, it remains unclear which NK subset secretes suppressive cytokines.

In this study, we demonstrated that the population of TGF-β^+^ NK cells was decreased in human PBMC and lymphoid tissues from mice with AD than in healthy control. We further found that TGF-β^+^ NK cells were largely included in CD1d^hi^PD-L1^hi^CD27^+^ NK cell subset. Of interest, AD severity was relieved after an adoptive transfer of CD1d^hi^PD-L1^hi^CD27^+^ NK cell subset in mice by inhibiting T_H_2 immunity.

## Methods

### Human TGF-β^+^ NK Cell Analysis

Patients were treated at the Department of Allergy, Allergy and Clinical Immunology Center, Cheju Halla General Hospital (Jeju, Korea) between October 2017 and May 2018. Subjects underwent blood tests and skin prick tests as described below and fulfilled the criteria of Hanifin and Rajka (1). The subjects were selected at random regardless of age or sex, and was classified based on the SCORAD index, the amount of IgE, and the number of eosinophils in the blood (**Table 1**). This study was approved by the Institutional Review Board of Jeju Halla General Hospital (approval number: CHH-2016-L13-01).

**Table 1:** Values are presented as mean ± SD.

Characteristics	Healthy control	AD patients	p value
Number	n=5	n=8	
SCORAD index	0	25.73±3.126	
IgE (IU/ml)	61.02±21.4	695.6±254	p=0.0785
Eosinophils/μl	66.64±6.997	564.4±146.5	p=0.0230

AD, atopic dermatitis; SCORAD, scoring atopic dermatitis.

### Induction of MC903-Mediated Atopic Dermatitis Model

C57BL/6 (8 to 10 weeks old) female mice were purchased from Orient Bio (Gyeonggi-do, Korea). For MC903 treatment, mice were painted with 2 nmol of MC903 (calcipotriol, Tocris Bioscience, Minneapolis, MN) in 20 μL of ethanol on both ear for 12 consecutive days. At 24 hours after treatment, mice were euthanized and their lymphoid tissues were isolated for flow cytometric analysis. All mice were housed in a pathogen-free facility at Konkuk University (Seoul, Korea). All animal experiments were approved by the Institutional Animal Care and Use Committee (IACUC) of Konkuk University.

### CD1d^hi^PD-L1^hi^CD27^+^ NKreg Subset Adoptive Transfer

Live splenic CD1d^hi^PD-L1^hi^CD27^+^NK1.1^+^ NK subset or CD1d^lo/−^PD-L1^lo/−^CD27^−^NK1.1^+^ NK subset were isolated with a FACSAria system (BD Bioscience, San Jose, CA, USA). The purity of these cells was more than 95%. For *in vivo* adoptive transfer, each NK subset (2 x 10^5^ cells/0.2 ml of PBS) was transferred intravenously into recipient mice at 24 hours before challenge with MC903 to induce atopic dermatitis. For the depletion of TGF-β, the mice were also injected intraperitoneally with 300 μg of anti-TGF-β mAb (1D11.16.8, Bio X Cell, West Lebanon, NH) or an isotype-matched control IgG1 every 3 days (on day 0, 3, 6, and 9) (35, 36).

### Flow Cytometric Analysis

Single-cell suspensions were isolated from the spleen, cLN, and ear. Especially, ear tissue-derived single-cell suspensions were dissociated using a gentleMACS dissociator (Miltenyi Biotec, Bergisch Gladbach, Germany). For the detection of intracellular cytokines or Foxp3 in cells isolated from each tissue, isolated cells were stimulated with phorbol 12-myristate 13-acetate (PMA, 50 ng/ml, Sigma Aldrich, St. Louis, MO, USA), ionomycin (500 ng/ml, Sigma Aldrich), and Brefeldin A (3 μg/ml, eBioscience, San Diego, CA, USA) for 4 hours. Prior to cell surface markers staining, Fcγ receptors were blocked with anti-CD16/CD32 mAbs (2.4G2, BD Biosciences). Conjugated and dead cells were excluded by analysis based on forward and side light scatter parameters and staining with a Zombie NIR™ Fixable Viability kit (Biolegend, San Diego, CA, USA). Antibodies against surface proteins including CD1d (1B1), CD2 (RM2-5), CD4(RM4-5), CD11b (M1/70), CD11c (N418), CD18 (M18/2), CD49b (DX5), CD62L (MEL-14), CD127 (A7R34), CD161b/c (PK136), CD244 (2B4), ICOS (C398.4A), NKG2D (CX5), NKp46 (29A1.4), MHCII (M5/114.15.2), and LAP (TW7-16B4) were obtained from eBioscience. Antibodies for CD3 (17A2), PD-L1 (10F.9G2), CD49a (HMα1), and CD127 (A7R34) were obtained from BioLegend (San Diego, CA, USA). An anti-CD27 (LG.3A10) antibody was purchased from BD Biosciences. Antibodies for intracellular staining of IL-4 (11B11), IL-10 (JES5-16E3), IL-13 (eBio13A), T-bet (eBio4B10), and Foxp3 (FJK-16s) and a fixation/permeabilization kit were bought from eBiosciences. Anti-Eomes (W17001A) and Anti-CD3 (17A2) antibodies were purchased from BioLegend. For flow cytometric analysis of human TGF-β^+^ NK cells, human subject-derived peripheral blood mononuclear cells (PBMCs) were isolated by density gradient separation using Ficoll-Paque (GE Healthcare). Anti-CD3 (HIT3a, eBioscience), CD56 (CMSSB, eBioscience), and TGF-β1 (9016, R&D Systems, Minneapolis, MN) antibodies were used. Cells were stimulated with PIB for 4 hours before analysis with a FACSCanto II flow cytometer (BD Bioscience) and FlowJo version 10 software (Tree Star, Ashland, OR, USA).

### Quantitative Real Time-PCR

Total RNAs were extracted from mouse splenic NK subsets or ear NK cells using an RNA isolation kit easy-BLUE (iNtRON Biotechnology, Gyeonggi, Korea). cDNA synthesis and real-time PCR were performed on a LightCycler^®^480 II using the LightCycler^®^ 480 SYBR green I master mix (Roche Diagnostics, Mannheim, Germany) according to the manufacturer’s instructions. PCR amplification of glyceraldehyde-3-phosphate dehydrogenase (GAPDH) as a housekeeping gene was performed for each sample for normalization between samples. The intensity of the expression of each gene was quantitated using a LightCycler^®^480 Software 1.5.0 (Roche Diagnostics). The following primers were used: *Tgfb1* (Forward: 5′-CACCATCCATGACATGAACC-3′, Reverse: 5′-TCATGTTGGACAACTGCTCC-3′); *Gapdh* (Forward: 5′-AATGCATCCTGCACCACCAA-3′, Reverse: 5′-GGAGGCATGTAGGCCATGAGGTC-3′).

### Statistical Analysis

Data are expressed as mean ± standard error of the mean (SEM) from three or more independent *in vitro* or *in vivo* experiments. All statistical analyses were performed with Student’s *t*-test or one-way analysis of variance (ANOVA) with Tukey’s *post hoc* test. Statistical significance (*p < 0.05 and **p < 0.01) was determined with a GraphPad Prism 7.0 software (GraphPad Inc., San Diego, CA, USA).

## Results

### Alteration of Population of TGF-β-Producing NK Cells in Mice With Atopic Dermatitis

**Figure 1:**
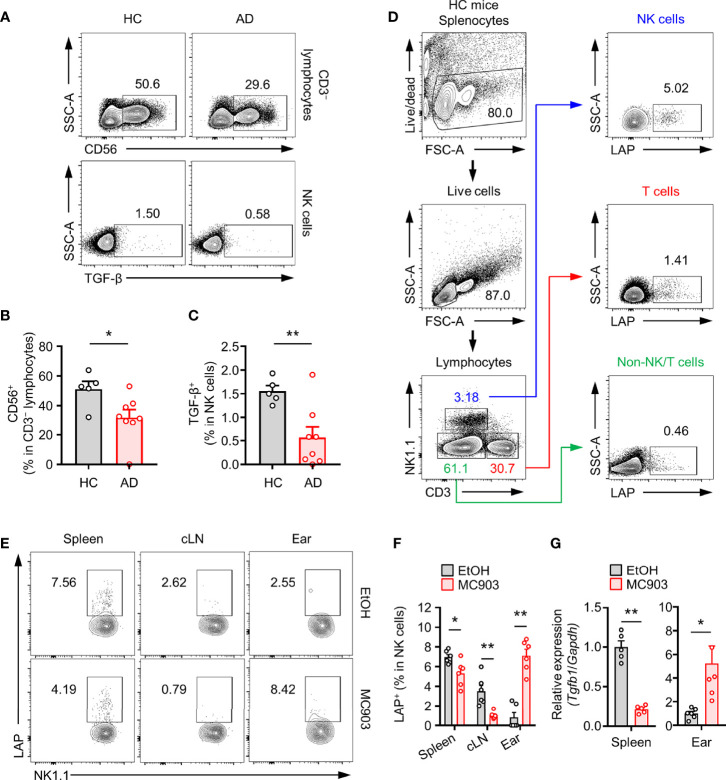
Population changes of TGF-β^+^ NK cells in an atopic dermatitis model. **(A)** Representative plot images showing TGF-β^+^CD56^+^CD3^–^ PBMCs from healthy controls (n = 5) or atopic dermatitis patients (n = 8). **(B)** Histograms showing frequencies of total NK cells and **(C)** TGF-β^+^ NK cells for panel **(A)**. **(D)** Representative flow cytometry images showing TGF-β^+^ leukocytes (NK cells, T cells, and Non-T/NK cells) in mouse spleen tissues. **(E)** Representative plot images showing LAP (latent TGF-β)^+^ NK cells in spleen, cLN, ear tissues from AD mouse model. **(F)** Histograms showing frequencies of TGF-β^+^ NK cells for panel E (n = 6). **(G)** Histograms showing gene expression of TGF-β isolated from splenic (n = 5) or ear (n = 6) NK cells. All values represent the mean ± SEM. *p < 0.05; **p < 0.01.

Accumulating evidences have indicated that NK cells are closely associated with AD progression in humans (37). We further investigated whether TGF-β^+^ NK cells might be involved in AD progression in humans through peripheral blood monocytes (PBMCs) from healthy controls and AD patients (**Table 1**). As reported, the population of total NK cells in PBMCs was decreased in AD patients than in healthy controls (**Figures 1A, B**). We also found that the population of TGF-β^+^ NK cells was reduced in PBMCs from AD patients (**Figure 1C**). To further characterize the potential role and mechanism of TGF-β^+^ NK cells to control AD symptom, we first checked population changes of TGF-β^+^ NK cells in normal mice (**Figure 1D**). We found that the frequency of splenic TGF-β1/latency associated peptide (LAP)^+^ (as TGF-β1) NK cells was higher in NK cells than in T cells or non-NK/T cells (**Figure 1D**). Interestingly, the frequency of LAP^+^ NK cells was decreased in spleen and cLN while the population of LAP^+^ NK cells was increased in ear tissues of mice with AD compared to that in normal mice (**Figures 1E, F**). We further observed that the expression of TGF-β mRNA from splenic or ear NK cells was changed in mice with AD compared to that in normal mice (**Figure 1G**). On the other hand, the population of LAP^+^CD3^+^ T cells did not show a significant change in spleen, cLN, and ear according to the development of AD (**Supplementary Figure 1**). Additionally, we also evaluated the expression of another anti-inflammatory cytokine, IL-10, but this also did not show any significant difference under normal or AD mice *in vivo* (**Supplementary Figure 2A**). Taken together, these results confirm that the tissue-specific population of LAP^+^ NK cells has a very closely associated with the disease development of AD mice.

### Identification of Surface Phenotype for TGF-β^+^ NK Cells

**Figure 2:**
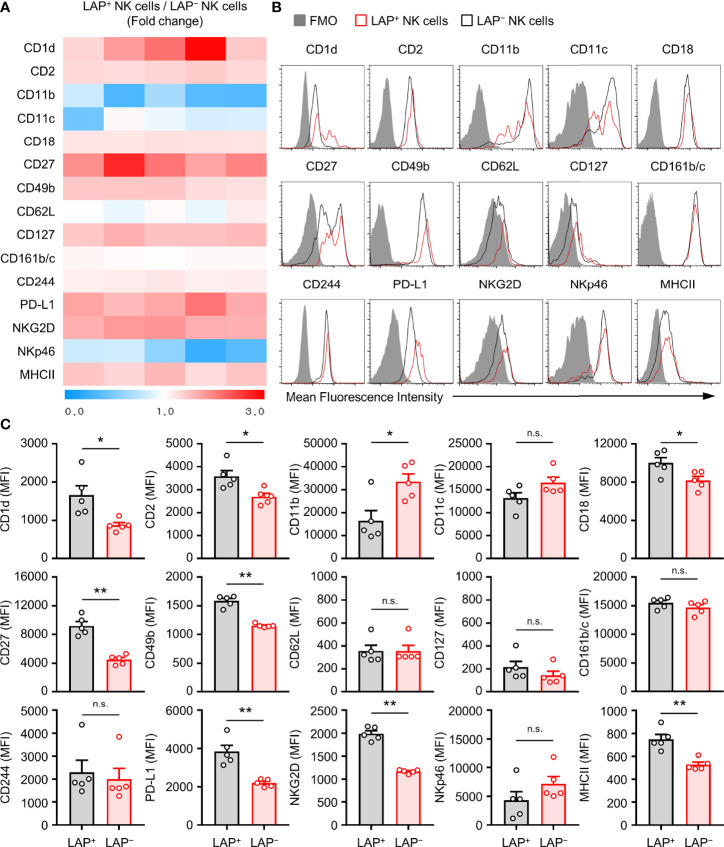
Characterization of TGF-β^+^ NK cells in mouse. **(A)** Heat map showing fold change of surface protein molecules expression between TGF-β^+^ and TGF-β^−^ splenic NK cells by flow cytometric analysis. The ratio values of LAP^+^/LAP^–^ NK protein mean fluorescence intensity (MFI) expression for each group were expressed from 0 to 3 folds (n = 5). **(B)** Representative histogram images for flow cytometric analysis of cell surface molecules of TGF-β^+^ and TGF-β^−^ NK cells. **(C)** Histograms showing the MFI of each surface molecules on NK cells. Results are expressed as representative images and the mean ± SEM from five independent experiments. *p < 0.05; **p < 0.01; n.s., not significant.

To find unique phenotypical surface makers of TGF-β^+^ NK cells, expression levels of potential NK cell surface makers in LAP^+^ and LAP^−^ splenic NK cells were compared. As shown in **Figure 2**, expression levels of CD1d, CD2, CD18, CD27, CD49b, PD-L1, NKG2D, and MHCII were increased in LAP^+^ NK cells than in LAP^−^ NK cells (**Figure 2**). It has been reported that the CD27^+^ NK cell subset is a unique subset for the production of effector cytokines and that CD11b^+^ NK cell subset has a strong cytotoxicity (38, 39). In our results, LAP^+^ NK cells showed high CD27 expression and low CD11b expression compared to LAP^−^ NK cells. Therefore, LAP^+^ NK cells might be capable of producing cytokines rather than causing cytotoxicity in NK subsets. In addition, we found that expression levels of CD1d and PD-L1 in LAP^+^ NK cells were higher than those in LAP^−^ NK cells (**Figure 2C**).

### TGF-β^+^ NK Cells Are Largely Included in CD1d^hi^PD-L1^hi^CD27^+^ NK Cell Subset

**Figure 3:**
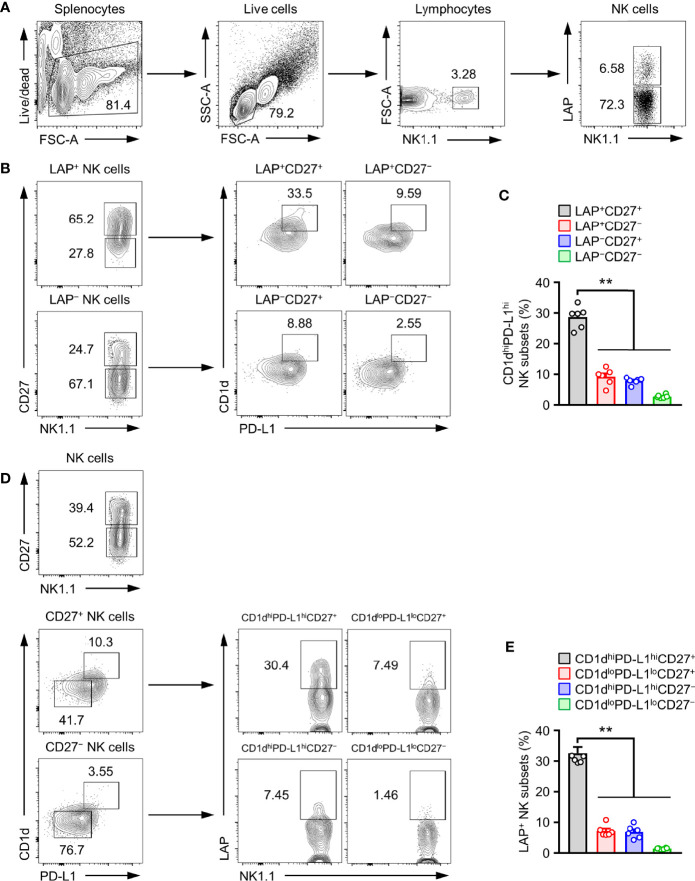
CD1d^hi^PD-L1^hi^CD27^+^ subset is TGF-β-Producing regulatory NK cells. **(A)** Gate strategies for LAP^+^ NK cells. **(B)** Representative flow cytometry images, **(C)** Histograms for the frequency of CD1d^hi^PD-L1^hi^ subsets in LAP^+^CD27^+^ NK cells (n = 6). **(D)** Representative flow cytometry images and **(E)** histograms for the frequency of LAP^+^ subsets in CD1d^hi^PD-L1^hi^CD27^+^ NK cells (n = 6). Data are expressed as mean ± SEM. **p < 0.01.

The above results prompted us to investigate whether the development of TGF-β^+^ NK cells might be associated with expression levels of CD1d, CD27, and PD-L1 on NK cells. CD27 was highly expressed on LAP^+^ NK cells than on LAP^−^ NK cells (**Figures 3A, B**). The population of CD1d^high^ and PD-L1^high^ NK cell subset was observed in 28.7 ± 1.5% of LAP^+^CD27^+^ NK cells (**Figure 3C**). Additionally, we analyzed the population of LAP^+^ NK cells in other NK cell subsets such as CD1d^hi^PD-L1^hi^CD27^+^NK, CD1d^lo^PD-L1^lo^CD27^+^NK, CD1d^hi^PD-L1^hi^CD27^-^NK, and CD1d^lo^PD-L1^lo^CD27^-^ NK subsets. Among them, the CD1d^hi^PD-L1^hi^CD27^+^NK subset had the highest frequency (32.6 ± 2.0%) of LAP^+^ NK cells in healthy control mice (**Figures 3D, E**). These results suggest that the expression of CD27, CD1d, and PD-L1 is closely associated with TGF-β production in NK cells.

### Populations of TGF-β^+^ NK Cells Are Correlated With the CD1d^hi^PD-L1^hi^CD27^+^ NK Subset in Peripheral Tissues

**Figure 4:**
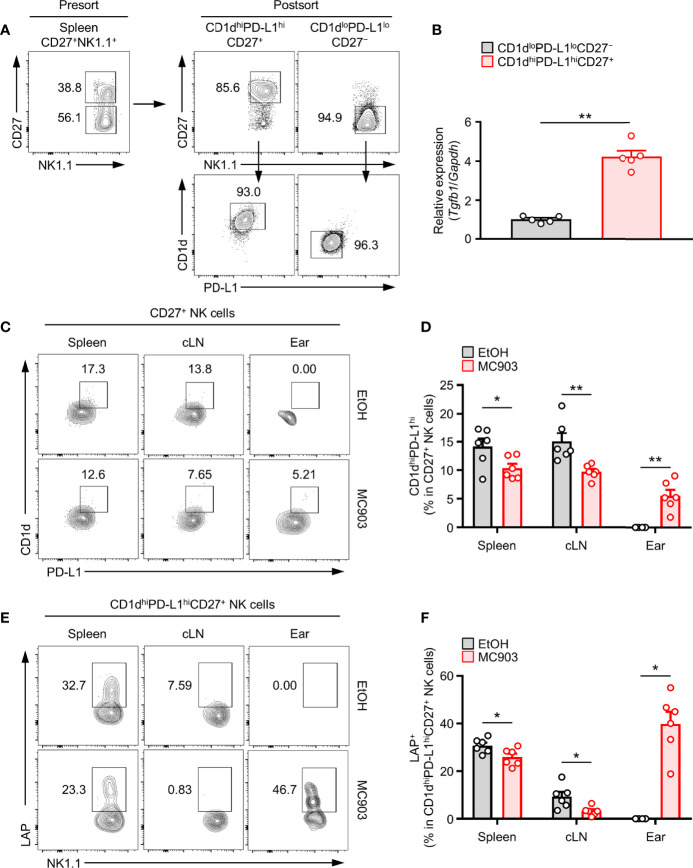
Correlation between TGF-β-Producing NK cells and CD1d^hi^PD-L1^hi^CD27^+^ NKreg subsets in an AD mouse model. **(A)** Splenic CD1d^hi^PD-L1^hi^CD27^+^ or CD1d^lo^PD-L1^lo^CD27^–^ NK subsets were isolated from mice by sorting with a FACSAria flow cytometer. Representative flow cytometry images and purities of sorted cells. **(B)** Histograms showing gene expression of TGF-β from the splenic CD1d^hi^PD-L1^hi^CD27^+^ or CD1d^lo^PD-L1^lo^CD27^–^ NK subsets (n = 5). **(C)** Representative flow cytometry images and **(D)** histograms for the frequency of CD1d^hi^PD-L1^hi^ subsets in LAP^+^CD27^+^ NK cells in spleen, cLN, and ear from AD mouse (n ≥ 6 per each group). **(E)** Representative flow cytometry images and **(F)** histograms for the frequency of LAP+ in CD1d^hi^PD-L1^hi^CD27^+^ NK cells in spleen, cLN, and ear from AD mouse (n ≥ 6 per each group). All values represent the mean ± SEM. *p < 0.05; **p < 0.01.

In the above phenotypical analysis, we found that CD1d^hi^PD-L1^hi^CD27^+^ NK subset had the highest expression than other NK subsets (**Figure 3**). Next, we checked how unique CD1d^hi^PD-L1^hi^CD27^+^ NK subsets were for TGF-β production compared to other NK subsets. In the experiment using CD1d^hi^PD-L1^hi^CD27^+^ and CD1d^lo^PD-L1^lo^CD27^−^ NK subsets (**Figure 4A**), we further found that the expression of TGF-β was much higher in CD1d^hi^PD-L1^hi^CD27^+^ NK subsets than in CD1d^lo^PD-L1^lo^CD27^−^ NK subsets (**Figure 4B**).

Next, we tested whether the expression of TGF-β in NK cells was associated with population change of CD1d^hi^PD-L1^hi^CD27^+^ NK subset in AD mouse model. Consistent with the above results (**Figures 1E, F**), we further discovered that the frequency and number of CD1d^hi^PD-L1^hi^CD27^+^ NK subsets were also decreased in the spleen and cLN but increased in ears of mice with AD (**Figures 4C–F**). As in the proportion of total TGF-β^+^ NK cells in **Figure 1F**, the proportion of CD1d^hi^PD-L1^hi^CD27^+^ NK subsets was also decreased in lymphoid tissues and increased in the target skin lesions. TGF-β production in CD1d^hi^PD-L1^hi^CD27^+^ NK subsets was also synergistically changed. These results indicate that population changes of TGF-β^+^ NK cells are closely associated with those of CD1d^hi^PD-L1^hi^CD27^+^ NK subsets in mice with AD. Like a **Supplementary Figure 2A**, we also compared the expression of IL-10, another anti-inflammatory cytokine, in the suggested CD1d^hi^PD-L1^hi^CD27^+^ NK subsets, but showed no AD-dependent changes in each lymphoid tissue (**Supplementary Figure 2B**). Altogether, these results suggest that the CD1d^hi^PD-L1^hi^CD27^+^ NK subset contains a large portion of TGF-β-producing NK subset.

### CD1d^hi^PD-L1^hi^CD27^+^ NK Subsets Suppress Symptoms of AD *via* Suppression of ILC2s in Mice

**Figure 5:**
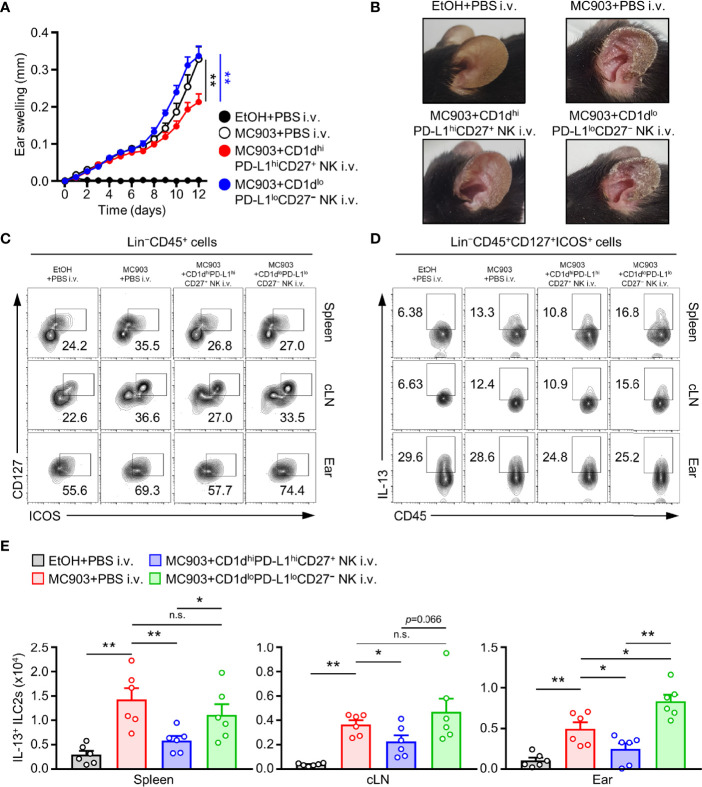
Adoptive transferred NKreg subsets inhibits IL-13^+^ ILC2s activity and atopic dermatitis responses. **(A)** Ear thicknesses and **(B)** Representative ear images in MC903-induced AD mice with or without the transfer of CD1d^hi^PD-L1^hi^CD27^+^ or CD1d^lo^PD-L1^lo^CD27^–^ NK subset. **(C)** Representative flow cytometry images for the frequency of Lin^–^CD45^+^CD127^+^ICOS^+^ (ILC2s), **(D)** IL-13^+^ ILC2s, and **(E)** histograms for the number of IL-13^+^ ILC2s in spleen, cLN, and ear from AD mice with or without transfer of CD1d^hi^PD-L1^hi^CD27^+^ or CD1d^lo^PD-L1^lo^CD27^–^ NK subset (n = 6). Data are expressed as mean ± SEM. *p < 0.05; **p < 0.01; n.s., not significant.

There are three types of innate lymphoid cells (ILCs) such as type 1, type 2, and type 3 ILCs (40, 41). Among them, type 2 ILC is well recognized to be able to induce allergic inflammation by secreting IL-4, IL-5, and IL-13 as by T_H_2 cells in various allergic responses (42–44). In particular, ILC2 has been accepted as a major effector cell type for the secretion of IL-5 and IL-13 in MC903-induced AD mice (8, 9). Hence, we checked whether CD1d^hi^PD-L1^hi^CD27^+^ NK subset could suppress the population of ILC2 in the AD mouse model. To test this, we adoptively transferred CD1d^hi^PD-L1^hi^CD27^+^ NK subsets or CD1d^lo^PD-L1^lo^CD27^−^ NK subsets as the control into MC903-induced AD mice. Adoptive transfer of CD1d^hi^PD-L1^hi^CD27^+^ NK subsets but not CD1d^lo^PD-L1^lo^CD27^−^ NK subsets largely suppressed symptoms of AD in mice (**Figure 5A**). The thickness for representing ear swelling was reduced largely by the transfer of CD1d^hi^PD-L1^hi^CD27^+^ NK subsets, but not by CD1d^lo^PD-L1^lo^CD27^−^ NK subsets (**Figure 5B**). These results suggest that TGF-β-producing CD1d^hi^PD-L1^hi^CD27^+^ NK subsets play a pivotal role in the inhibition of MC903-induced AD symptoms. Besides, adoptive transfer of CD1d^hi^PD-L1^hi^CD27^+^ NK subsets but not CD1d^lo^PD-L1^lo^CD27^−^ NK subsets significantly inhibited numbers of IL-13^+^ ILC2s in spleen, cLN, and ear tissues of AD mice (**Figures 5C-E**). In more detail, these results showed that although the distribution of ILC2 in each tissue was suppressed by administration of CD1d^hi^PD-L1^hi^CD27^+^ NK subsets (**Figure 5C**), IL-13 expression in ILC2 was not restricted (**Figure 5D**). These results suggested that the adoptive transfer effect of the CD1d^hi^PD-L1^hi^CD27^+^ NK subset resulted from the suppression of the increase in the number of IL-13^+^ ILC2 in peripheral tissues of mice with AD (**Figure 5E**).

### CD1d^hi^PD-L1^hi^CD27^+^ NK Subsets Suppress the Development of Atopic Dermatitis Through Inhibition of T_H_2 and Maintenance of Treg in T Cell Immunity

**Figure 6:**
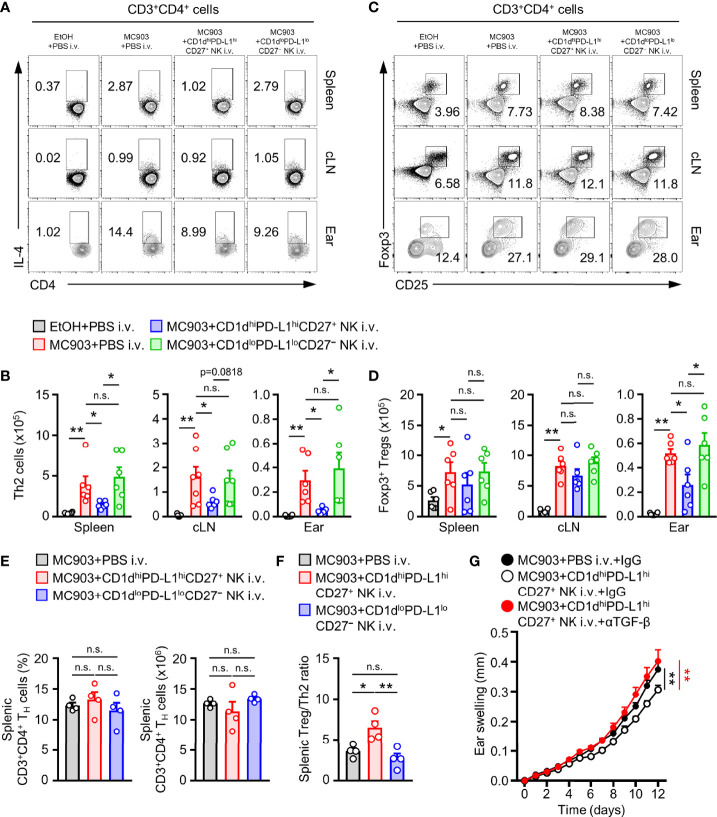
Adoptive transferred TGF- β-producing NK subsets suppresses T_H_2 response and helps balance between effector and regulatory T cells. **(A)** Representative flow cytometry images and **(B)** histograms for the number of T_H_2 cells in spleen, cLN, and ear from AD mice after adoptive transfer CD1d^hi^PD-L1^hi^CD27^+^ or CD1d^lo^PD-L1^lo^CD27^–^ NK subset (n = 6). **(C)** Representative flow cytometry images and **(D)** histograms for the number of T_H_2 cells in spleen, cLN, and ear from AD mice (n = 6). **(E)** Histograms for the frequency and number of total T_H_ cells in spleen from AD mice with or without transfer of CD1d^hi^PD-L1^hi^CD27^+^ or CD1d^lo^PD-L1^lo^CD27^–^ NK subset. **(F)** Histograms showing the ratio of Foxp3^+^ Treg cells per IL-4^+^ T_H_2 cells from spleen tissues as indicated (n = 4). Data are expressed as mean ± SEM. *p < 0.05; **p < 0.01; n.s., not significant. **(G)** The ear thickness of CD1d^hi^PD-L1^hi^CD27^+^ NK subset transferred MC903-induced AD mice with or without anti-TGF-β mAb treatment are shown (n = 7). The results are expressed as the mean ± SEM from two independent experiments. **P < 0.01 versus MC903+PBS i.v.+IgG (black**) or MC903+CD1d^hi^PD-L1^hi^CD27^+^ NK i.v.+αTGF-β mAb (red**).

To determine how CD1d^hi^PD-L1^hi^CD27^+^ NK subsets affected the development of AD, we observed changes in the population of IL-4^+^ T_H_2 and Foxp3^+^ Tregs cell in CD1d^hi^PD-L1^hi^CD27^+^ NK subsets transferred AD mice. We found that the number of T_H_2 cells was significantly reduced in CD1d^hi^PD-L1^hi^CD27^+^ NK subsets transferred AD mice compared to that in CD1d^lo^PD-L1^lo^CD27^−^ NK subsets transferred AD mice (**Figures 6A, B**). It is generally accepted that TGF-β can induce Treg cells activity in murine and human (45). Thus, we further tested whether the effect of adoptive transferred CD1d^hi^PD-L1^hi^CD27^+^ NK subsets influenced the change of Treg cell population. Results showed that adoptive transfer of CD1d^hi^PD-L1^hi^CD27^+^ NK subsets did not cause any change in the number of Treg cells in spleen or cLN (**Figure 6B**). However, reduced number of Treg cells was observed in ear tissues after adoptive transfer of CD1d^hi^PD-L1^hi^CD27^+^ NK subsets (**Figures 6C, D**) probably caused by a decrease in total CD4^+^ T_H_ cells in skin lesions. It seems to be a phenomenon that the infiltration of immune cells into the target tissue is reduced as much as the reduction of AD aggravation. We also found that the population and number of spleen-derived CD4^+^ T_H_ cells were not different (**Figure 6E**). However, the ratio of Treg cells per effector T_H_2 cells was much higher after the adoptive transfer of CD1d^hi^PD-L1^hi^CD27^+^ NK subsets compared to that of CD1d^lo^PD-L1^lo^CD27^−^ NK subsets (**Figure 6F**). Furthermore, we applied a TGF-β neutralizing antibody to AD mice to check whether the inhibitory effect of the CD1d^hi^PD-L1^hi^CD27^+^ NK subsets was indeed TGF-β-dependent *in vivo*. When the TGF-β neutralizing antibody was treated, the inhibitory effect of the adoptively transferred NK subsets was restored in the ear swelling of the MC903-induced AD mouse model (**Figure 6G**). Altogether, these results suggest that CD1d^hi^PD-L1^hi^CD27^+^ NK subsets can suppress T_H_2 cells development in MC903-induced AD mouse model and affect the balance of inflammatory or regulatory T cells. And it was confirmed that it was TGF-β-dependent to limit the exacerbation of AD mouse model.

## Discussion

NK cells are well recognized as innate immune cells that participate in various immune responses to viral infections or cancers by exhibiting anti-viral or anti-tumor effects (46–48). Although NK cells are lymphocytes, like other types of innate lymphoid cells (ILCs), they are generally involved in both innate immunity and adaptive immunity. NK cells are fundamental immune cells that play important roles in the initiation of immune responses and body homeostasis (49). They usually secrete signature cytokine such as IFN-γ. They are involved in the formation of T_H_1 immunity in the body. They are known to play a crucial role in eliminating infected or tumor cells through secretion of intrinsic digestive enzymes (50, 51). Previous studies have shown that the frequency of NK cells in the body is generally decreased in various cancer diseases. This trend is considered to be due to the immune escape mechanism of cancer cells from host immune surveillance (52, 53). Recent studies have reported that NK cells are decreased in PBMCs from AD patients (34). This trend predicts that maintaining the balance of the number of NK cells and the immune circumstance will be important for the pathogenesis of allergy diseases such as AD. Our results also showed that the frequency of NK cells (CD56^+^CD3^−^) in the blood of AD patients was reduced (**Figures 1A, B**). Previous studies have found that NK cells are decreased in the blood of atopic dermatitis patients but increased in AD lesions, suggesting that NK cells can migrate to control peripheral T_H_2 responses (34). However, the definite cause and mechanism of the alteration of NK cells in AD remain unclear. Therefore, we focused on another perspective to understand NK cells in atopic dermatitis. In addition to the well-known classical function, NK cells are known to have a subset of regulatory functions like other immune cells (14, 54).

In the past, researchers have paid attention to the etiological aspect caused by changes in the distribution and activity of a subset of specific immune cells due to breakdown of the balance of immune state in the body. In the 2000s, it was found that some sub-phenotypes of immune cells, including regulatory T cells (Tregs), were regulatory subsets that could restore the body’s immune status to normal through immunomodulation or induction of immune tolerance (55). Previous studies have shown that T cells are Tregs, B cells are regulatory B cells (Bregs), monocyte/neutrophils are myeloid-derived suppressor cells (MDSCs), macrophages are M2 or alternative macrophages, dendritic cells (DCs) are tolerogenic DCs, and ILCs are regulatory ILCs. Thus, a regulatory phenotype has been reported for each immune cell (56–61). These regulatory immune cells can differentiate to have their own characteristic functions by secreting anti-inflammatory cytokines such as IL-10 and TGF-β to control pro-inflammatory cells (62).

It is also known that NK cells have a regulatory type (called NKreg or NK3) (14). In addition to dividing effector functions into NK1 or NK2 cells according to inflammatory types, it has been proposed that secreting IL-10 or TGF-β can control excessive inflammatory responses (27, 28). It has been reported that NK cells can secrete anti-inflammatory cytokines such as IL-10 and TGF-β through secretion profiling, although there are only a small population of NK cells in the whole body. Especially, IL-10-producing NK cells are known to control the activation of T cells while Prf1^−/−^ mice-derived IL-10^+^ NK cells regulate CD8^+^ T cells and contribute to immunological maintenance in mouse cytomegalovirus (MCMV) infection (25, 26). In human studies, a regulatory NK subset that can secrete IL-10 and TGF-β has been reported (26, 27). It has been confirmed that IL-10 and TGF-β-producing NK cells exist in peripheral blood mononuclear cells and decidua in pregnant women. In particular, TGF-β-producing NK cells are significantly increased in decidua. It has been suggested that these regulatory NK cells contribute to pregnancy tolerance (26, 27). The present study showed that TGF-β-producing NK cells were present in human PBMCs. It also revealed that not only NK cells, but also TGF-β^+^ NK cells were decreased in allergic patients (**Figure 1C**). We employed MC903, a representative atopic dermatitis mouse model, to find changes in TGF-β^+^ NK cells in atopic dermatitis mice. Although there was a difference in the total number of lymphocytes, interestingly, TGF-β expression was higher in NK cells than in other lymphocytes of mouse spleen. Thus, we could predict that NK cells are an important source of TGF-β (**Figure 1D**). After MC903 treatment, TGF-β^+^ NK cells were significantly reduced in mouse spleen and cervical LN (cLN), a draining lymph node (LN), whereas these cells were increased in ear tissues, a peripheral target site of the disease (**Figures 1E–G**). As mentioned above, these patterns are similar to the migration pathway of classical IFN-γ^+^ NK cells in atopic dermatitis (34).

Previous studies have predicted that NK cells also have a regulatory type that can secrete anti-inflammatory cytokines such as IL-10 and TGF-β and that they are expected to be involved in immune tolerance or regulation (14). However, elucidation of the characteristic phenotype of NKreg is still insufficient. Through this study, we analyzed the expression of major receptors in mouse TGF-β^+^ NK cells (**Figure 2**) and found high TGF-β expression (32.6 ± 2.0%) in CD1d^hi^PD-L1^hi^CD27^+^ NK subsets (**Figure 3**). However, it is necessary to evaluate whether our proposed TGF-beta-producing NK subsets overlap with T cells, NKT cells, γδT cells or helper ILC1s. Therefore, as a result of the confirmation, it can be seen that the TGF-beta-producing NK subsets is CD3^−^CD49a^−^CD49b^+^Eomes^+^T-bet^+^ conventional NK cells (**Supplementary Figure 3**).

We proposed that these highly TGF-β-expressing NK cells were splenic CD1d^hi^PD-L1^hi^CD27^+^ subsets. Several human NK cell studies have suggested an antigen-presenting role of NK cells. CD1d is a non-polymorphic, MHC class I-like molecule (63, 64). It is well known that CD1d usually presents antigens such as glycolipids, including α-galactosylceramide (α-GC), to CD1d-restricted NKT cells (65). CD1d is mostly expressed in innate immune cells such as DC, macrophages, B cells, and ILCs. In our study, it was confirmed that CD1d expression of TGF-β^+^ NK cells was high than TGF-β^−^ NK cells (66, 67). PD-L1 is well known to be expressed in NK cells as a representative immunosuppression marker (68). The murine CD27 expressing NK cell is exhibits potent cytokine production and high migratory capacity (39). Their correlation with the distribution pattern of TGF-β-producing NK subsets in AD was also determined. As a result, with AD development, CD1d^hi^PD-L1^hi^CD27^+^ NK subsets were decreased in lymphoid organs such as spleen and cLN, but markedly increased in the ear (**Figure 4**). Based on these results, we can explain that the CD1d^hi^PD-L1^hi^CD27^+^NK1.1^+^CD3^−^ phenotype in mice is due to TGF-β-producing regulatory NK subsets. In addition, the distribution of CD1d^hi^PD-L1^hi^CD27^+^ NK subsets was decreased in spleen and draining LN from AD mouse model, suggesting that this distribution might be increased in skin lesions where type 2 inflammation appears.

This study not only suggested a phenotype of TGF-β^+^ NK cells, but also confirmed that the proposed TGF-β^+^CD1d^hi^PD-L1^hi^CD27^+^ NK subsets could regulate type 2 inflammation like AD. To demonstrate the immunomodulatory effect of TGF-β-producing CD1d^hi^PD-L1^hi^CD27^+^ NK subset *in vivo*, we separated CD1d^hi^PD-L1^hi^CD27^+^ NK subsets and CD1d^lo^PD-L1^lo^CD27^−^ NK subsets and adaptively transferred before MC903 induction of AD using a mouse model. Compared to vehicle mice (MC903+PBS i.v.) and CD1d^lo^PD-L1^lo^CD27^−^ NK subsets transferred mice, significant inhibitory effects were observed in CD1d^hi^PD-L1^hi^CD27^+^ NK subsets (**Figures 5A, B**).

Type 2 innate lymphoid cells (ILC2s) have recently been proposed as important effector cells in allergic responses. They are involved in peripheral allergic conditions through secretion of IL-4, IL-5, and IL-13 (42–44). We tested how CD1d^hi^PD-L1^hi^CD27^+^ NK subsets could affect the activity of ILC2 in the AD mouse model and found a decrease of systemic ILC2 (**Figures 5C–E**).

AD is well known as a typical chronic allergic disease. The classical immune system is initiated as allergen, resulting in a hypersensitivity reaction mediated by innate immune cells followed by a T_H_2-cell mediated chronic inflammatory response (4). It is difficult to adequately overcome atopic dermatitis by controlling only the initial hypersensitivity reaction by innate immune cells. Thus, we tested how TGF-β-producing CD1d^hi^PD-L1^hi^CD27^+^ NK subsets could affect the activity of T_H_2 effector cells in AD through *in vivo* adaptive transfer. Results confirmed that the addition of TGF-β-producing NK subsets induced a decrease in T_H_2 cells without controlling the number of total T cells in lymphoid organs, thereby blocking the activity of T cells polarizing with T_H_2 (**Figure 6**).

It is well known that TGF-β can induce T cells into Foxp3^+^ regulatory T cells (iTregs) among several immunomodulatory functions (36, 69). In addition, Treg cells are the most representative immunomodulatory cells known to regulate various allergic disorders such as atopic dermatitis through several studies (70, 71). Therefore, we considered whether the control of T_H_2-mediated inflammatory responses such as ILC2s and T_H_2 cells by CD1d^hi^PD-L1^hi^CD27^+^ NK subsets could be correlated with Foxp3^+^ Treg cells. In our results, CD1d^hi^PD-L1^hi^CD27^+^ NK subsets did not directly control the number of Foxp3^+^ Treg cells in lymphoid tissues compared to its inhibitory effect on T_H_2-mediated inflammatory cells. On the other hand, the number of Foxp3^+^ Treg cells was decreased in in the ear from the CD1d^hi^PD-L1^hi^CD27^+^ NK-treated group (**Figures 6C, D**). It was found that Foxp3^+^ Treg cells were maintained by inhibiting T_H_2 cells activity without changing the distribution or the number of CD4^+^ T cells in the spleen. Therefore, administration of CD1d^hi^PD-L1^hi^CD27^+^ NK subsets in an AD-induced state can increase the ratio of Foxp3^+^ Treg cells compared to PBS control (**Figures 6E, F**). The decrease of Foxp3^+^ Treg cells in CD1d^hi^PD-L1^hi^CD27^+^ NK subsets administered ear appeared to result in reduced infiltration of CD4^+^ cells in ears. It can be seen that the disease improvement effect of CD1d^hi^PD-L1^hi^CD27^+^ NK subsets administration lies in the inhibition of infiltration of peripheral CD4^+^ T cells rather than the induction of an increase of peripheral Treg cells. According to this, CD1d^hi^PD-L1^hi^CD27^+^ NK subsets mainly controls T cell-mediated inflammatory responses in lymphoid tissues. Thus, treatment with CD1d^hi^PD-L1^hi^CD27^+^ NK subsets decreased T_H_2 but increased the ratio of Treg cells in the spleen and draining lymph node.

In conclusion, we found that CD1d^hi^PD-L1^hi^CD27^+^ was a unique TGF-β-producing NK subset and that treating such regulatory subset in a mouse AD disease model inhibited T_H_2-mediated effector cells and helped improve disease exacerbation. Although more diverse mechanism studies and mutual evaluation in human studies are needed, results of this study suggest that NK cell-derived regulatory subset can be used in various ways as a novel immune disease treatment strategy.

## Data Availability Statement

The original contributions presented in the study are included in the article/**Supplementary Material**. Further inquiries can be directed to the corresponding authors.

## Ethics Statement

The studies involving human subjects were reviewed and approved by the Institutional Review Board of Jeju Halla General Hospital (CHH-2016-L13-01). The patients/participants provided their written informed consent to participate in this study. The animal experiments were approved by the Institutional Animal Care and Use Committee (IACUC) of Konkuk University.

## Author Contributions

HK and WC designed the experiments, analyzed the data, and wrote the paper. KM and JK performed most of the experiments. GN analyzed the human sample data. DL and MJ collected and analyzed flow cytometry data. JL, MK, and SH performed *in vivo* experiments. All authors contributed to the article and approved the submitted version.

## Funding

This research was supported by the National Research Foundation of Korea (NRF) grant funded by the Korea government (NRF-2020R1C1C1003676 and NRF-2021R1A2B5B03002157).

## Conflict of Interest

The authors declare that the research was conducted in the absence of any commercial or financial relationships that could be construed as a potential conflict of interest.

## Publisher’s Note

All claims expressed in this article are solely those of the authors and do not necessarily represent those of their affiliated organizations, or those of the publisher, the editors and the reviewers. Any product that may be evaluated in this article, or claim that may be made by its manufacturer, is not guaranteed or endorsed by the publisher.
