# Supplementary figures and images for: CD1dhiPD-L1hiCD27+ Regulatory Natural Killer Subset Suppresses Atopic Dermatitis
Source: Front Immunol. 2022 Jan 5;12:752888. doi: 10.3389/fimmu.2021.752888 (PMC8766675; doi:10.3389/fimmu.2021.752888)

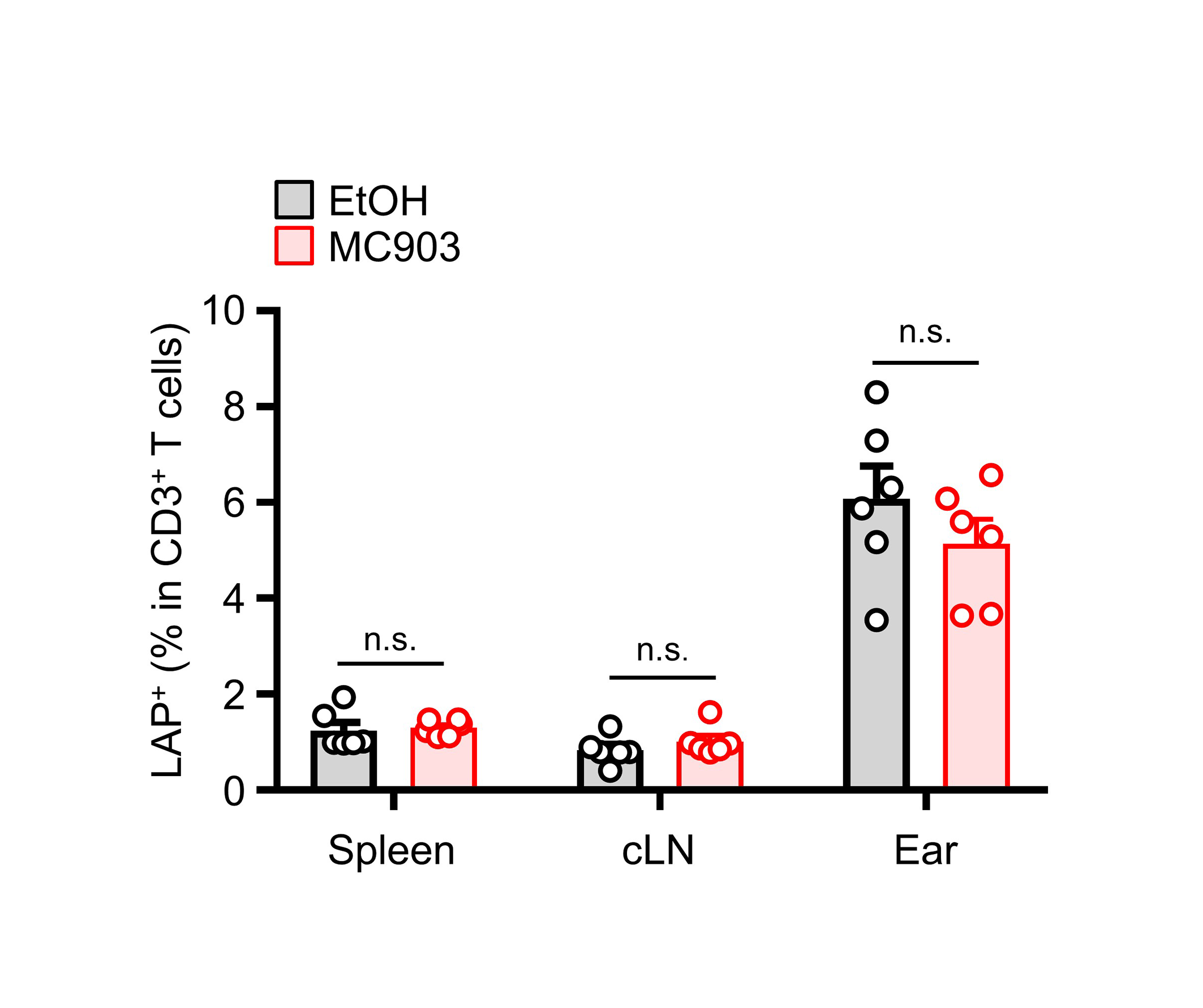

Supplement: Supplementary file 1 [file Image_1.tif]

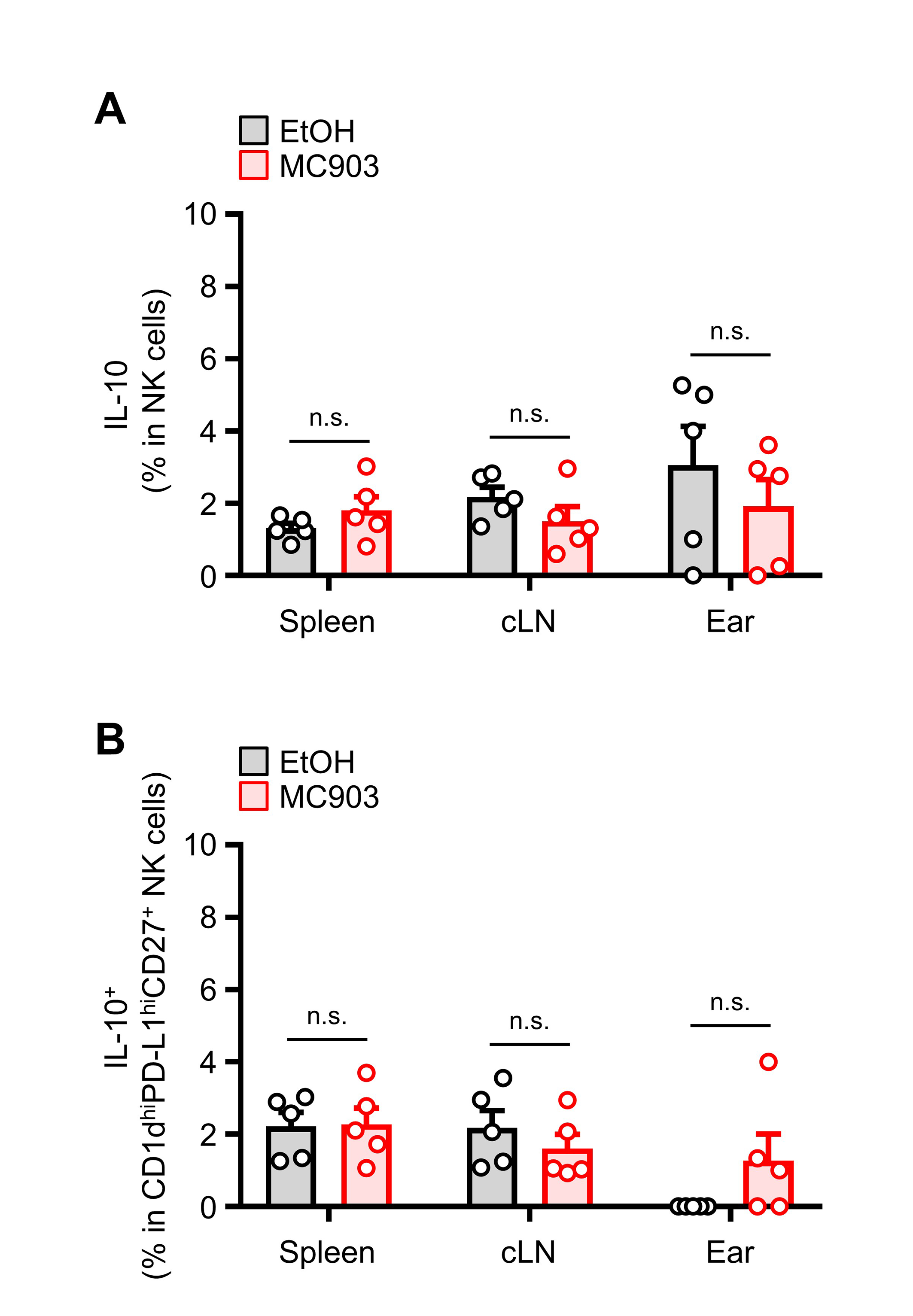

Supplement: Supplementary file 2 [file Image_2.tif]

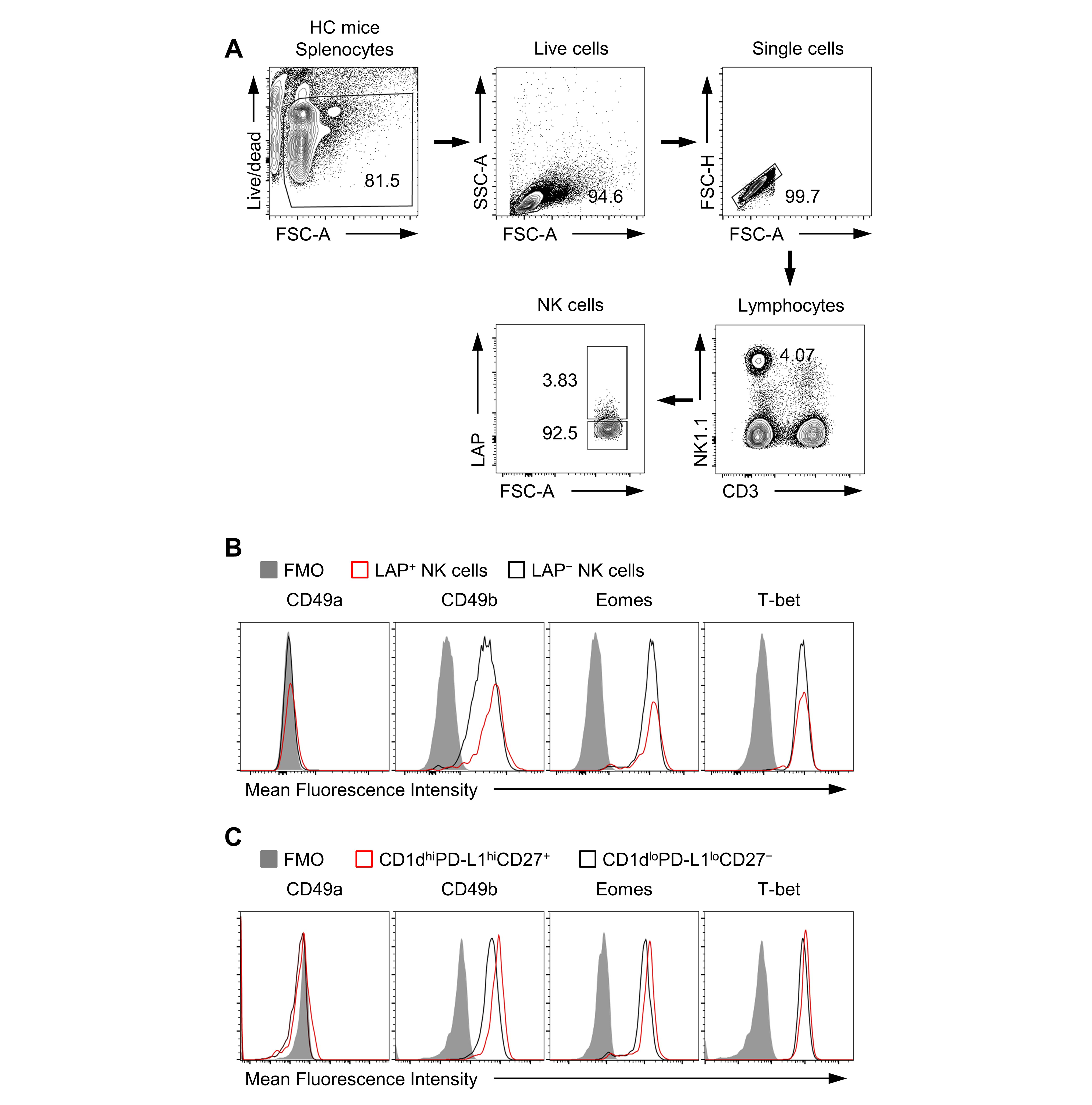

Supplement: Supplementary file 3 [file Image_3.tif]
